# A comparative genomics approach reveals a local genetic signature of Leishmania tropica in Morocco

**DOI:** 10.1099/mgen.0.001230

**Published:** 2024-04-05

**Authors:** Hasnaa Talimi, Othmane Daoui, Giovanni Bussotti, Idris Mhaidi, Anne Boland, Jean-François Deleuze, Rachida Fissoune, Meryem Lemrani, Gerald F. Späth

**Affiliations:** 1 Laboratory of Parasitology and Vector-Borne-Diseases, Institut Pasteur du Maroc, Casablanca, Morocco; 2 Systems and Data Engineering Team, National School of Applied Sciences, University Abdelmalek Essaadi, Tangier, Morocco; 3 Institut Pasteur, Université Paris Cité, INSERM 1201, Unité de Parasitologie Moléculaire et Signalisation, Paris, France; 4 Université Paris-Saclay, CEA, Centre National de Recherche en Génomique Humaine (CNRGH), 91057, Evry, France

**Keywords:** cutaneous leishmaniasis, GIP, *Leishmania tropica*, Morocco, NGS

## Abstract

In Morocco, cutaneous leishmaniasis (CL) caused by *Leishmania* (*L.*) *tropica* is an important health problem. Despite the high incidence of CL in the country, the genomic heterogeneity of these parasites is still incompletely understood. In this study, we sequenced the genomes of 14 Moroccan isolates of *L. tropica* collected from confirmed cases of CL to investigate their genomic heterogeneity. Comparative genomics analyses were conducted by applying the recently established Genome Instability Pipeline (GIP), which allowed us to conduct phylogenomic and principal components analyses (PCA), and to assess genomic variations at the levels of the karyotype, gene copy number, single nucleotide polymorphisms (SNPs) and small insertions/deletions (INDELs) variants. Read-depth analyses revealed a mostly disomic karyotype, with the exception of the stable tetrasomy of chromosome 31. In contrast, we identified important gene copy number variations across all isolates, which affect known virulence genes and thus were probably selected in the field. SNP-based cluster analysis of the 14 isolates revealed a core group of 12 strains that formed a tight cluster and shared 45.1 % (87 751) of SNPs, as well as two strains (M3015, Ltr_16) that clustered separately from each other and the core group, suggesting the circulation of genetically highly diverse strains in Morocco. Phylogenetic analysis, which compared our 14 *L. tropica* isolates against 40 published genomes of *L. tropica* from a diverse array of locations, confirmed the genetic difference of our Moroccan isolates from all other isolates examined. In conclusion, our results indicate potential regional variations in SNP profiles that may differentiate Moroccan *L. tropica* from other *L. tropica* strains circulating in endemic countries in the Middle East. Our report paves the way for future research with a larger number of strains that will allow correlation of diverse phenotypes (resistance to treatments, virulence) and origins (geography, host species, year of isolation) to defined genomic signals such as gene copy number variations or SNP profiles that may represent interesting biomarker candidates

**Impact Statement**

Comparative analysis of 14 novel genomes from Moroccan isolates of *Leishmania tropica* collected from confirmed cutaneous leishmaniasis cases using our recently established Genome Instability Pipeline (GIP, Späth and Bussotti, *Nucleic Acid Research* 2022) identified a core group of 12 isolates that were genetically highly related but evolutionarily distant to the current * L. tropica* reference genome. We further uncovered two highly divergent strains, M3015 and Ltr_16, that were phylogenetically distinct from each other as well as from the core group. Together our results suggest the presence of a SNP profile that distinguishes Moroccan *L. tropica* isolates from those found in other endemic regions. Our results pave the way for future genomic research with a larger number of strains across various endemic regions to further corroborate geographical adaptation of this important pathogen. Our work thus establishes the experimental framework for future biomarker discovery that will correlate defined genomic signals to diverse phenotypes (resistance to treatments, virulence) and origins (geography, host species, year of isolation).

## Data Summary

The genome sequences of the 13 *Leishmania tropica* isolates FJ2002, FJ2004, FJ2005, FJ2007, FJ2008 FJ2010, FJ2011, FJ2012, M1314, M2007, M2013, M3015 and M2571 sequenced in this study were deposited in the National Center for Biotechnology Information (NCBI) database under the BioSample accessions SAMN35562195, SAMN35562196, SAMN35562197, SAMN35562198, SAMN35562199, SAMN35562200, SAMN35562201, SAMN35562202, SAMN35562203, SAMN35562204, SAMN35562205, SAMN35562206 and SAMN35562207, respectively. The BioProject ID is PRJNA978932. In addition, Ltr_16 is publicly available under accession number SAMN08162952 (BioProject: PRJNA422027).

## Introduction

Leishmaniasis is a vector-borne disease of global reach with important public health impact. The disease is of zoonotic or anthroponotic origin and is caused by protozoan parasites of the genus *Leishmania* that show cutaneous or visceral tropisms. The parasite is transmitted by the bites of infected female phlebotomine sandflies, which feed on blood [1]. Cutaneous leishmaniasis (CL) is the most frequent form of the disease, associated with skin lesions, primarily ulcers, on exposed regions of the body, resulting in life-long scars that can cause social stigma [2]. The Americas, the Mediterranean Basin, the Middle East and Central Asia account for around 95 % of all CL cases. In 2020, ten countries accounted for more than 85 % of new CL cases: Afghanistan, Algeria, Brazil, Colombia, Iraq, Libya, Pakistan, Peru, the Syrian Arab Republic and Tunisia. Every year, between 6 00 000 and 1 million new CL cases are reported globally [3]. The epidemiology of leishmaniasis is determined by both parasite and sandfly species, local ecological factors of transmission locations such as animal reservoir or climatic conditions, present and previous parasite exposure of the human population, and human behaviour [3]. Approximately 70 mammal species, including humans, have been identified as natural reservoir hosts of *Leishmania* parasites [3].

In Morocco, CL is caused by three *Leishmania* species, namely *L. major*, *L. infantum* and *L. tropica*. The last is the main causative agent of CL due to its wide geographical distribution and high frequency, as judged by the appearance of new foci [4]. CL caused by *L. tropica* was described for the first time in 1987 in a child who had resided in the province of Azilal, central Morocco [5]. Thereafter, a large hypo-endemic focus between Tadla and Agadir has been identified [6]. Since the mid-1990s, CL outbreaks due to *L. tropica* have occurred in emerging foci in central and northern Morocco, even in areas previously known for *L. major* transmission [4,7, 8]. *L. tropica* is a highly diverse species, especially in Morocco, as previously demonstrated by analysis of zymodemes, which refers to a classification system based on enzyme electrophoresis patterns, used to distinguish strains. Indeed, Morocco is home to the highest number of *L. tropica* zymodemes identified in North Africa from human CL patients, dogs and sandflies (i.e. *L. tropica* MON-102, MON-107, MON-109, MON-112, MON-113, MON-122, MON-123 and MON-279) [6,9]. This contrasts with findings from Tunisia and Libya where only *L. tropica* MON-8 (syn *Leishmania killicki*) was identified, and its closest neighbour Algeria, where *L. tropica* MON-301 (related to *L. killicki*) was identified [10]. This *L. tropica* polymorphism in Morocco was also revealed by RAPD, PCR-RFLP of the antigen-encoding genes *gp63* and *cpb*, sequence analysis of intergenic spacer regions (ITS), multilocus microsatellite typing (MLMT) and multilocus sequence typing (MLST) [11,12]. However, despite numerous studies supporting the diversity of *L. tropica* within the Moroccan territory and with respect to other endemic countries, the genetic heterogeneity has not been addressed yet on a genome-wide level.

Here, we address this limitation by performing comparative genomics analyses with 14 novel genomes of *L. tropica* that were isolated from confirmed cases of CL in Morocco, with the aim to gain first insight into the genome diversity of *L. tropica* within Morocco and with respect to other endemic countries. We used the Genome Instability Pipeline (GIP) [13] to assess differences in karyotype, gene copy number, SNPs and small insertion/deletion (INDEL) variants.

## Methods

### Ethics considerations

All human adult participants provided informed consent prior to their involvement. For the inclusion of young children, consent was obtained from their respective parents or legal guardians. The study underwent thorough evaluation and received approval from the Ethical Review Committee for Biomedical Research (CERB) of the Faculty of Medicine and Pharmacy, Rabat, Morocco (IORG 0006594 FWA 00024287), adhering to established research guidelines.

In the absence of a national Moroccan Ethics committee for animal experimentation, parasite isolation from dogs was carried out in accordance with the Institut Pasteur of Morocco recommendations and guidelines, which comply with (i) the Charter of Fundamental Rights of the European Union (2000/C 364/01, https://www.europarl.europa.eu/charter/pdf/text_en.pdf), and (ii) the Council Directive 86/609/EEC of 24 November 1986 on ‘The Approximation of Laws, Regulations And Administrative Provisions Of The Member States Regarding The Protection Of Animals Used For Experimental And Other Scientific Purposes (http://data.europa.eu/eli/dir/1986/609/oj)’.

### *Leishmania* isolates and parasite culture

**Fig. 1.**
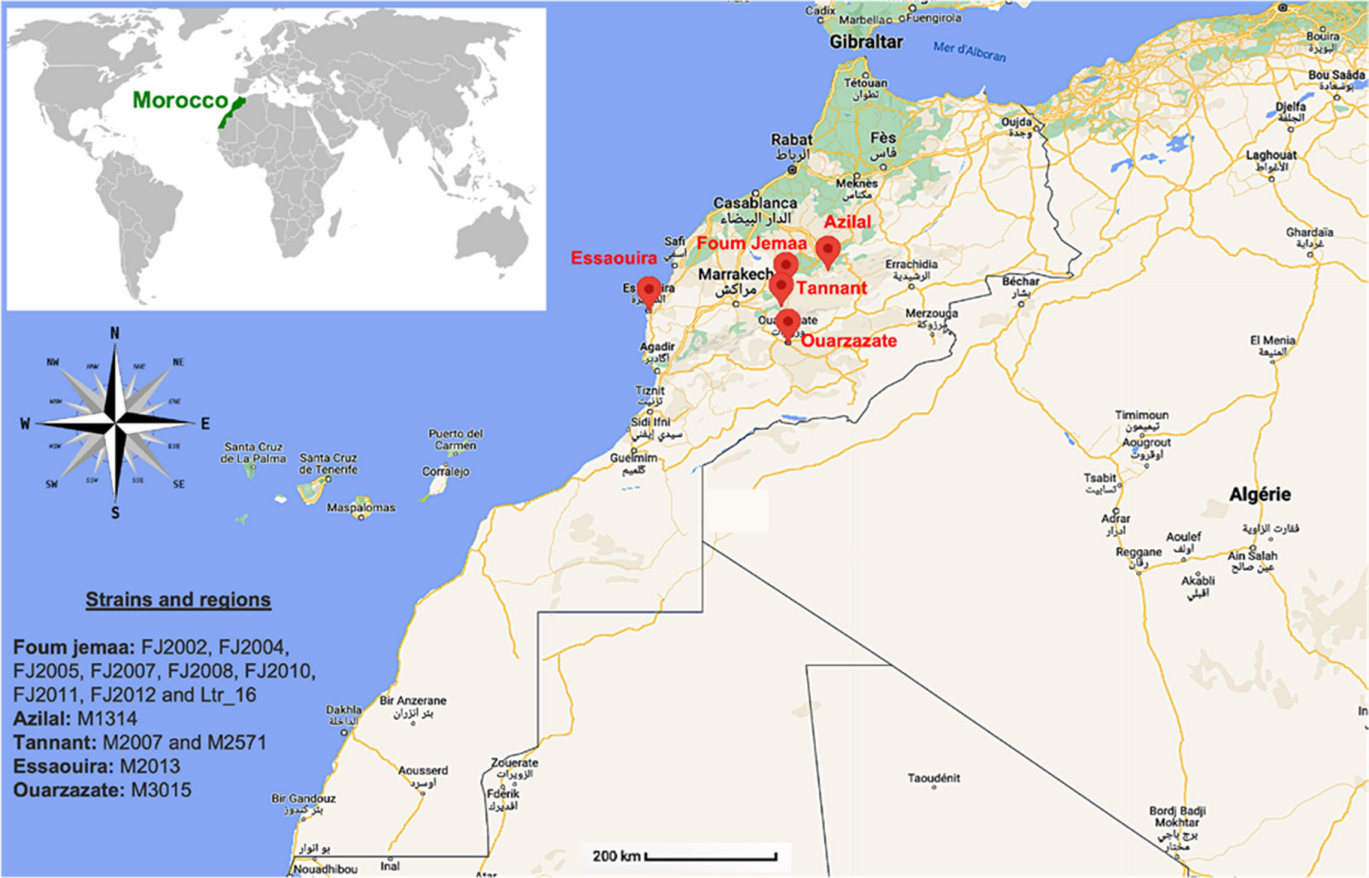
Map showing the geographical origin of the isolates used in our study.

We included in this study 14 isolates of *L. tropica* from confirmed cases of CL in Morocco, which were collected from 13 patients and one dog between 1990 and 2020 in various regions of Morocco, including Essaouira, Ouarzazate and the macrofoci of the Azilal province (Foum Jemaa, Azilal and Tanant) (Table 1 and Fig. 1).

**Table 1.** Overview of isolates

Sample ID	Host/pathology	Origin	Year of isolation	Specimen	Zymodeme	Molecular typing	Passage	Circumstances of sampling
FJ2002	Human/CL	Foum Jemaa	2020	Skin tissue	nd	ITS1-PCR-RFLP	≤5	Cured
FJ2004	Human/CL	Foum Jemaa	2020	Skin tissue	nd	ITS1-PCR-RFLP	≤5	Cured
FJ2005	Human/CL	Foum Jemaa	2020	Skin tissue	nd	ITS1-PCR-RFLP	≤5	nd
FJ2007	Human/CL	Foum Jemaa	2020	Skin tissue	nd	ITS1-PCR-RFLP	≤5	Relapse
FJ2008	Human/CL	Foum Jemaa	2020	Skin tissue	nd	ITS1-PCR-RFLP	≤5	Cured
FJ2010	Human/CL	Foum Jemaa	2020	Skin tissue	nd	ITS1-PCR-RFLP	≤5	Cured
FJ2011	Human/CL	Foum Jemaa	2020	Skin tissue	nd	ITS1-PCR-RFLP	≤5	nd
FJ2012	Human/CL	Foum Jemaa	2020	Skin tissue	nd	ITS1-PCR-RFLP	≤5	Cured
Ltr_16	Human/CL	Foum Jemaa	2016	Skin tissue	nd	ITS1-PCR-RFLP	≤5	Cured
M1314	Human/CL	Azilal	1988	Skin tissue	MON-109	ITS1-PCR-RFLP	nd	nd
M2007	Canine/CanL	Tannant	1990	Skin tissue	MON-102	ITS1-PCR-RFLP	nd	nd
M2013	Human/CL	Essaouira	1990	Skin tissue	MON-102	ITS1-PCR-RFLP	nd	nd
M3015	Human/CL	Ouarzazate	1995	Skin tissue	MON-264	ITS1-PCR-RFLP	nd	nd
M2571	Human/CL	Tannant	1993	Skin tissue	MON-102	ITS1-PCR-RFLP	nd	nd

CanL Canine Leishmaniasis

Sampling of isolates with identifiers beginning with ‘FJ’ (FJ2002, FJ2004, FJ2005, FJ2007, FJ2008, FJ2010, FJ2011 and FJ2012) were collected from Foum Jemaa, a specific geographical location in Morocco (see Fig. 1). Parasites were isolated by dermal scraping of the lesion edge. Isolates were first grown in biphasic NNN (Novy–MacNeal–Nicolle) medium at 25 °C and established cultures were expanded in RPMI 1640 (Biowest) medium supplemented with 2 mM l-glutamine (Eurobio), 15 % FBS (Biowest) and 1 % penicillin/streptomycin (100 U ml^−1^ penicillin and 100 µg ml^−1^ streptomycin; Biowest). Cultures were maintained for a maximum of five passages prior to analysis to limit genomic change caused by long-term culture adaptation [14,15]. The Ltr_16 isolate was obtained in 2016 from a human case of CL in Foum Jemaa. The sample was taken by dermal scraping of the periphery of the lesion.

Isolates with identifiers starting with ‘M’ (M1314, M2007, M2013, M2571 and M3015) were received from the Montpellier University Hospital Center (CHU, Parasitology-Mycology laboratory, site Antonin Balmes-La Colombière). These *L. tropica* strains were obtained from skin lesions of human patients and one infected dog and cultured on NNN medium. To ensure their long-term preservation, the strains were cryopreserved in liquid nitrogen at the Mycology-Parasitology laboratory in Montpellier. Upon our request, the strains were thawed and subsequently cultured on NNN medium. Following their arrival in our laboratory, the strains underwent two to five passages. Subsequently, the parasites were collected from the cultures to extract DNA for further analysis.

### DNA extraction and sequencing analysis

Cryopreserved *Leishmania* promastigotes were thawed and first cultured at 25 °C in NNN medium before being grown in RPMI 1640 medium (Biowest). DNA extraction was performed using a QIAamp DNA Mini Kit (Qiagen) according to the manufacturer’s instructions. DNA quality and quantity were assessed using NanoDrop (Thermo Fisher Scientific).

Whole genome sequencing (WGS) was performed by the Centre National de Recherche en Génomique Humaine (CNRGH, Institut de Biologie François Jacob, CEA, Evry, France). After quality control, genomic DNA (1 µg) was used to prepare the library for WGS on an automated platform, using the Illumina ‘TruSeq DNA PCR-Free Library Preparation Kit’, according to the manufacturer’s instructions. After normalization and quality control, qualified libraries were sequenced on a HiSeqX5 platform (Illumina), as paired-end, 150 bp reads. In total, 24 samples were pooled on one lane of the HiSeqX5 flow cell. Sequence quality parameters were assessed throughout the sequencing run and standard bioinformatics analysis of sequencing data was based on the Illumina pipeline to generate a FASTQ file for each sample. The WGS data for Ltr16 were generated as described in Bussotti *et al*. [14] by the Institut Pasteur Biomics sequencing platform (https://research.pasteur.fr/en/team/biomics/) with Hiseq 2500 rapid runs.

### GIP and giptools

All results presented in this study were generated using the GIP [13], version 1.0.9. The GIP code is maintained and freely distributed at the GitHub page under https://github.com/giovannibussotti/GIP. All GIP outputs were used as input for giptools, a suite of 13 modules that allows the comparison of GIP-processed samples and visualization of changes in chromosomal copy number, gene copy number and variants (SNP/INDEL). Complete documentation for GIP and giptools, including a description of all options, is available at https://gip.readthedocs.io/en/latest/.

### Read alignment

WGS reads were mapped by GIP using BWA-MEM (version 0.7.17) [16] run with option -M, and the ‘Leishmania_tropica_CDC216-162’ reference genome [17]. BAM files were assessed for mapping quality by examining flagstat files and coverage was calculated with SAMtools (version 1.17) [18] to determine the average mapped read depth across the whole genome. Read duplicates were removed using Picard MarkDuplicates (http://broadinstitute.github.io/picard) (version 2.18.9) with the option ‘VALIDATION_STRINGENCY=LENIENT’. The ‘giptools overview’ module was run to gather the alignment statistics as estimated by Picard CollectAlignmentSummaryMetrics.

### Sequencing coverage ratio

For each sample, we started by loading files containing bin sequencing coverage data. To ensure accurate comparisons, we normalized the coverage values for each genomic bin. The normalization process included calculating the ratio of the actual coverage at a given bin to the median coverage across all bins. These steps were performed using BEDTools (version 2.31.0) [19] in conjunction with R (tidyverse package). The coverage ratio between the two isolates FJ2002 and M3015 was calculated by using the giptools ‘binCNV’ module.

### SNP and INDEL detection

SNPs and INDELs were called and extracted using Freebayes (version 1.3.2) [20], then GATK ‘SelectVariants’ (version 4.0.0.0) [21] was used to distinguish between them. Evaluation of the impact of these variants on genomic characteristics and potential functional consequences was conducted using SNPEff (version 4.3 t). SNPEff relies on a genome annotation file in gff3 file format, which is generated using the Companion software package (http://companion.gla.ac.uk/), an online server explicitly designed for genome annotation and analysis [22]. The identified SNPs were categorized based on their impact, to distinguish between high- and low-impact variations. High-impact variations denote alterations that are likely to have substantial consequences on protein structure or function, potentially leading to notable phenotypic changes. In contrast, low-impact variations represent changes that are less likely to cause major disruptions, often occurring in non-coding regions or having milder effects on protein function. To assess the number of unique and shared SNPs between all isolates, we generated an UpSet plot using the ‘UpSetR’ package. We visualized only 63 intersections out of 6765, selecting the most important ones for our analysis. All 6765 intersections are listed in Table S5 (available in the online version of this article). This allowed us to calculate the total number of SNPs recorded in the 14 isolates by adding up all the numbers listed in Table S5.

Additionally, to enhance our understanding of the distribution patterns, a kernel density analysis was applied using the giptools ‘SNV’ module, providing the position and the variant allele frequency of detected SNPs across the 36 chromosomes for M3015, Ltr_16, FJ2005 and M2013 samples (Fig. S1).

### Phylogenomic analyses

Phylogenomic tree reconstruction involved multiple steps to ensure robustness and accuracy. Initially, high-quality SNP variant call files (VCFs) were processed and combined into a master VCF using BCFtools (version 1.17). The unified VCF underwent conversion to Phylip format by employing the vcf2phylip.py Python script, available at https://github.com/edgardomortiz/vcf2phylip. We then utilized the advanced capabilities of IQ-TREE (version 2.2.6) [23] with 1000 bootstrap replicates to deduce the phylogenomic tree. This process generated several critical outputs, including the IQ-TREE report, maximum-likelihood tree and likelihood distances. The final stage entailed the graphical representation and colour enhancement of the phylogenomic tree through the interactive ITOL web platform [24], found at https://itol.embl.de/.

## Results

### Moroccan *L. tropica* isolates show karyotypic variations

Fourteen strains of *L. tropica* from confirmed CL cases were isolated from the Moroccan regions indicated in Fig. 1, expanded in culture for up to five passages to limit genomic change, and DNA was subjected to sequencing analysis (see Methods). The sequencing data were aligned against the reference genome ‘Leishmania_tropica_CDC216-162’ [17], allowing us to map 85.24–97.17 % of the reads at a depth of 60–154× (Table S1).

**Fig. 2.**
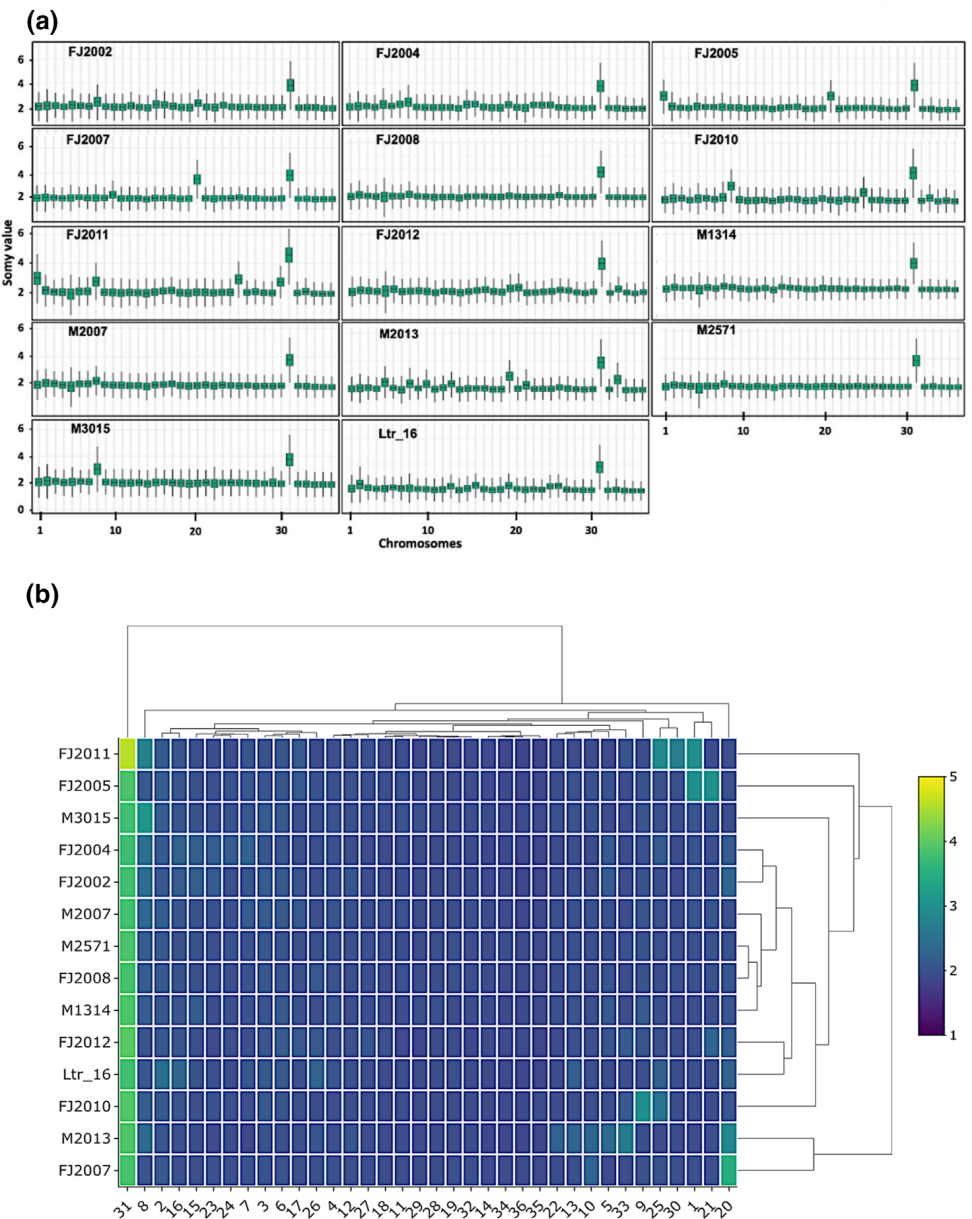
Assessment of the karyotypic profile. (**a**) Box plots representing the somy score distributions for each chromosome in the indicated samples. (**b**) Heatmap showing the estimated chromosomal somy score for each chromosome and isolate. The colour corresponds to the somy score as indicated in the key. The dendrogram identifies distinct clusters based on aneuploidy pattern and was constructed using Euclidean distances.

Comparison of chromosome numbers (somy scores) between isolates revealed a largely disomic karyotype for most of the isolates (somy score close to 2, Fig. 2a), with the following exceptions. First, as previously observed in all *Leishmania* species [15,25,27], chromosome 31 was tetrasomic in all isolates (somy score close to 4), except for FJ2011 showing a pentasomic state (somy score close to 5). Second, trisomic states (somy score of 3) were observed for various chromosomes in different isolates, e.g. chr 1 in FJ2005 and FJ2011, chr 8 in FJ2011 and M3015, chr 20 in FJ2007, or chr 21 in FJ2005 (Fig. 2a). The somy scores for some other chromosomes fell within the range 2–2.5 (e.g. chromosomes 5, 8 and 10 in M2013), indicating the presence of mosaic aneuploidies, which are probably attributed to the coexistence of cell sub-populations with varying somy scores, as previously described in *Leishmania donovani* [15].

We next analysed the 14 samples by hierarchical clustering based on their somy patterns (Fig. 2b). The resulting dendrogram identified four distinct clusters of isolates: (i) a cluster comprising M2013 (1990) and FJ2007 (2020) that share a trisomy of chromosome 20; and (ii) three clusters containing respectively FJ2012 (2020) and Ltr_16 (2016), FJ2004 (2020) and FJ2002 (2020), and M2007 (1990), M1314 (1988), M2571 (1993) and FJ2008 (2020), which seem to be defined by a similar pattern of mosaic aneuploidies. The remaining isolates (FJ2005, FJ2010, FJ2011 and M3015) are on separate branches due to their different karyotypic profile compared to the other isolates. Together these data demonstrate a level of genome instability in *L. tropica* that is similar to other *Leishmania* species [14,27]. Whether these karyotypic changes were selected in the field or are the result of culture adaptation remains to be established.

### Analysis of copy number variations

**Fig. 3.**
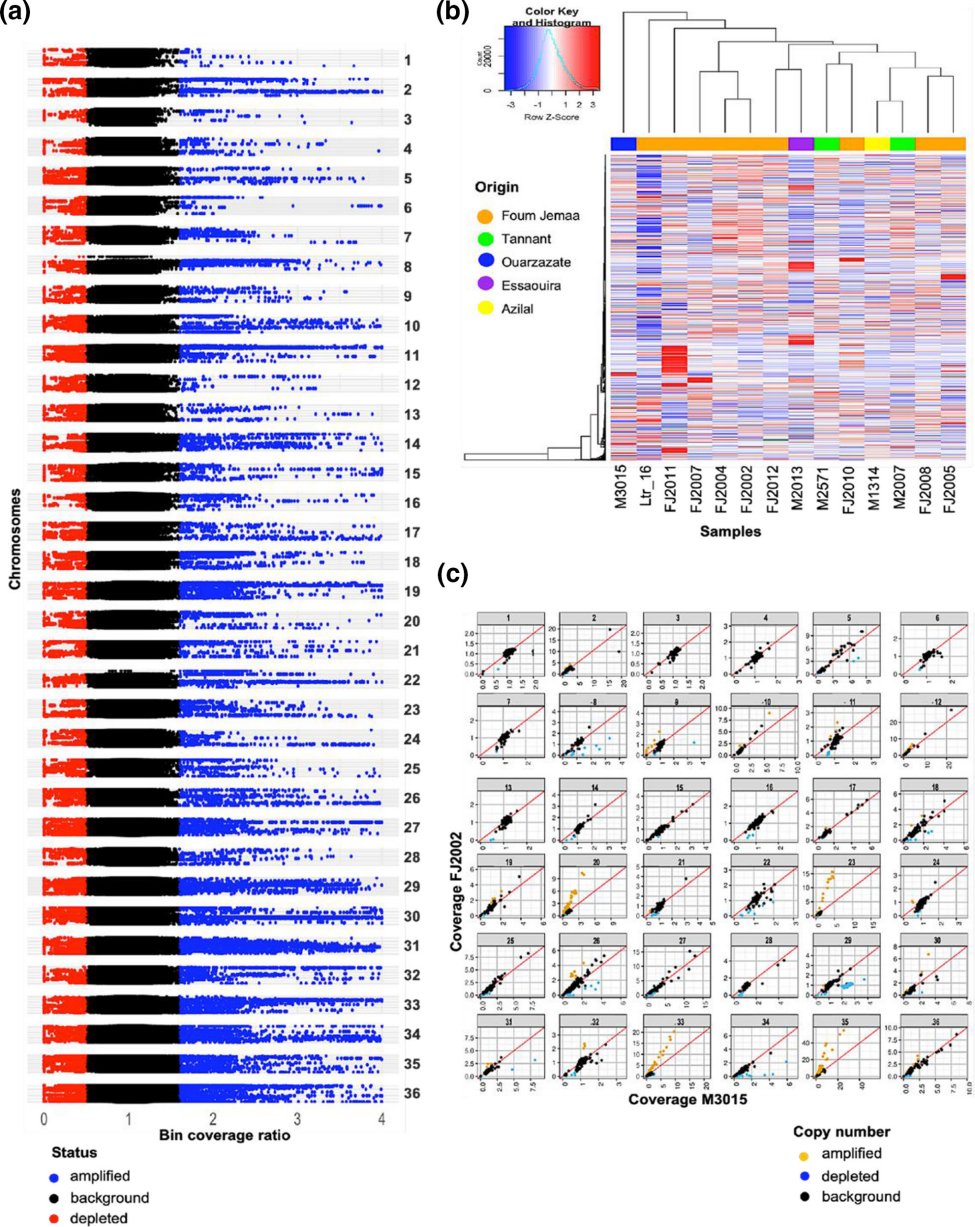
Copy number variation (CNV) analysis. (**a**) Manhattan plot. The normalized genomic bin sequencing coverage ratios (*x*-axis) of overlapping, 300 bp genomic bins (*x*-axis) are plotted against genomic position (*y*-axis) for each chromosome and all samples. Genomic regions with ratio scores >1.5 (compared to the reference genome) are highlighted in blue, while those with scores <0.75 are shown in red. (**b**) Gene CNV heatmap. The columns and the rows report respectively the samples and the detected gene CNVs. The colour scale indicates the normalized sequencing coverage of the genes. (**c**) Chromosome-specific scatter plots showing normalized sequencing coverage of genes in FJ2002 (*y*-axis) and M3015 (*x*-axis). Individual genes are represented by dots. Yellow dots, amplifications; blue dots, depletions; black dots, no notable change. The red diagonal lines indicate the bisectors.

We next assessed copy number variations (CNVs) by employing two complementary approaches. First, we used a bin-based approach that allowed us to monitor CNVs across the entire genome irrespective of its coding potential. Briefly, we calculated the mean coverage per chromosome, which served as the reference value against which we computed the coverage ratio for each bin (Fig. 3a and Table S2). This analysis allowed us to reveal important structural changes and genetic diversity between the isolates. From a total of 1 031 810 bins, we detected 2 175 amplified bins (2.1 % of the genome) showing a coverage increase of 1.5-fold or more compared to the average, and 1965 depleted regions (1.9 % of the genome) showing a coverage decrease of 0.75-fold or lower. Upon closer examination, these CNVs were not evenly distributed across the chromosomes but enriched in certain areas, suggesting possible hotspots of genome instability.

Second, we applied a gene-centred approach with the aim to assess biological functions that may be under selection in the field. The heatmap in Fig. 3(b) shows the normalized mean coverage of genes across all samples. Notably, despite their geographical proximity, the Foum Jemaa samples exhibit noteworthy gene CNVs, as reflected by their diverse distribution across distinct clusters. FJ2011 branches separately probably due to the large number of gene amplifications observed for this isolate. M3015 and Ltr_16 stand out as the most genetically diverse isolates, each forming distinctive branches on the heatmap. Ltr_16 seems to have undergone substantial reduction of gene copy number, suggesting that not only gene amplification but also gene depletion may be under selection, even though other explanations such as genetic drift cannot be ruled out (Fig. 3b).

To further investigate the breadth of gene CNVs in the highly diverse strain M3015, coverage ratios were calculated with respect to the Leishmania_tropica_CDC216-162 reference and plotted against FJ2002 as a benchmark, representing the genetically least diverse strain. This analysis revealed three distinct groups of genes: (i) genes whose copy number is centred around a ratio of 1 and thus similar to the reference genome in both strains; (ii) genes whose copy number ratios are close to the diagonal at values higher or lower than 1, indicating gene CNV differences to the reference genome that are shared between the two isolates; and (iii) genes with a copy number ratio above or below the diagonal, revealing gene CNVs between these two isolates (Fig. 3c). This last group included various genes that have been previously associated with virulence and other essential biological processes (Table S3). For M3015, for example, we revealed gene amplification for (i) PSA2 (Promastigote Surface Antigen Protein 2, LtrCDC216_12.0740), which showed a 1.5-fold increased read depth in M3015 and has been linked to *Leishmania* virulence and host immunomodulation [28], or (ii) the ATP-Dependent Clp Protease Subunit HSP78 (Heat Shock Protein 78, LtrCDC216_02.0140), which has been linked to parasite infectivity [29]. Conversely, gene depletion was observed in M3015 for (i) CPA (Cysteine Peptidase A, LtrCDC216_25.1015), which showed a 0.75-fold read depth and has been associated with parasite proteolytic activities [30], or (ii) an Amastin-Like Protein (LtrCDC216_08.0820), which has been linked to *Leishmania* survival within host macrophages [31] (Table S3).

In conclusion, our findings document important alterations in genome structure and CNV affecting both the intergenic and coding parts of the *L. tropica* genome. While karyotypic changes have been previously linked to short-term adaptation to culture, variations in gene CNVs are only selected during long-term culture (>20 passages, see Bussotti *et al*. [32]). Thus, the gene coverage differences we revealed in the 14 isolates probably reflect the genetic heterogeneity observed in the field, which may influence the virulence and infectivity of *L. tropica* isolates, shedding light on parasite adaptation strategies and their potential impact on disease outcomes.

### Comparative analyses of nucleotide variations

We next assessed the diversity of the Moroccan *L. tropica* isolates by analysing the number, frequency and localization of INDELs and SNPs. The analysis revealed a total number of SNPs per isolate ranging from 113 122 (M2007) to 1 50 676 (Ltr_16), while the number of INDELs ranged from 116 268 (Ltr_16) to 165 455 (M2571). Intriguingly, a substantial proportion of the SNPs were found in genic regions, ranging from 70 791 (M2007) to 86 935 (M3015). The percentage of non-synonymous SNPs, when considering all SNPs, ranges from 25.27 % (Ltr_16) to 29.01 % (M3015), suggesting potentially important functional and phenotypic differences in the two diverse isolates. Moreover, the percentage of high-impact SNPs that are predicted to affect protein function or structure ranged from 6.5 % (M1314 and M2007) to 7.8 % (Ltr_16). In contrast, the percentage of low-impact SNPs ranged from 29.28 % (Ltr_16) to 37.16 % (M3015), indicating changes that may have more subtle or moderate effects (as detailed in Table S4). Finally, all SNPs in the M3015, Ltr_16, FJ2005 and M2013 isolates show a high frequency above 90 % (Fig. S1A), with an additional small peak at 50 % observed for the diverse isolate M3015, which may indicate a previous hybridization event (Fig. S1B).

**Fig. 4.**
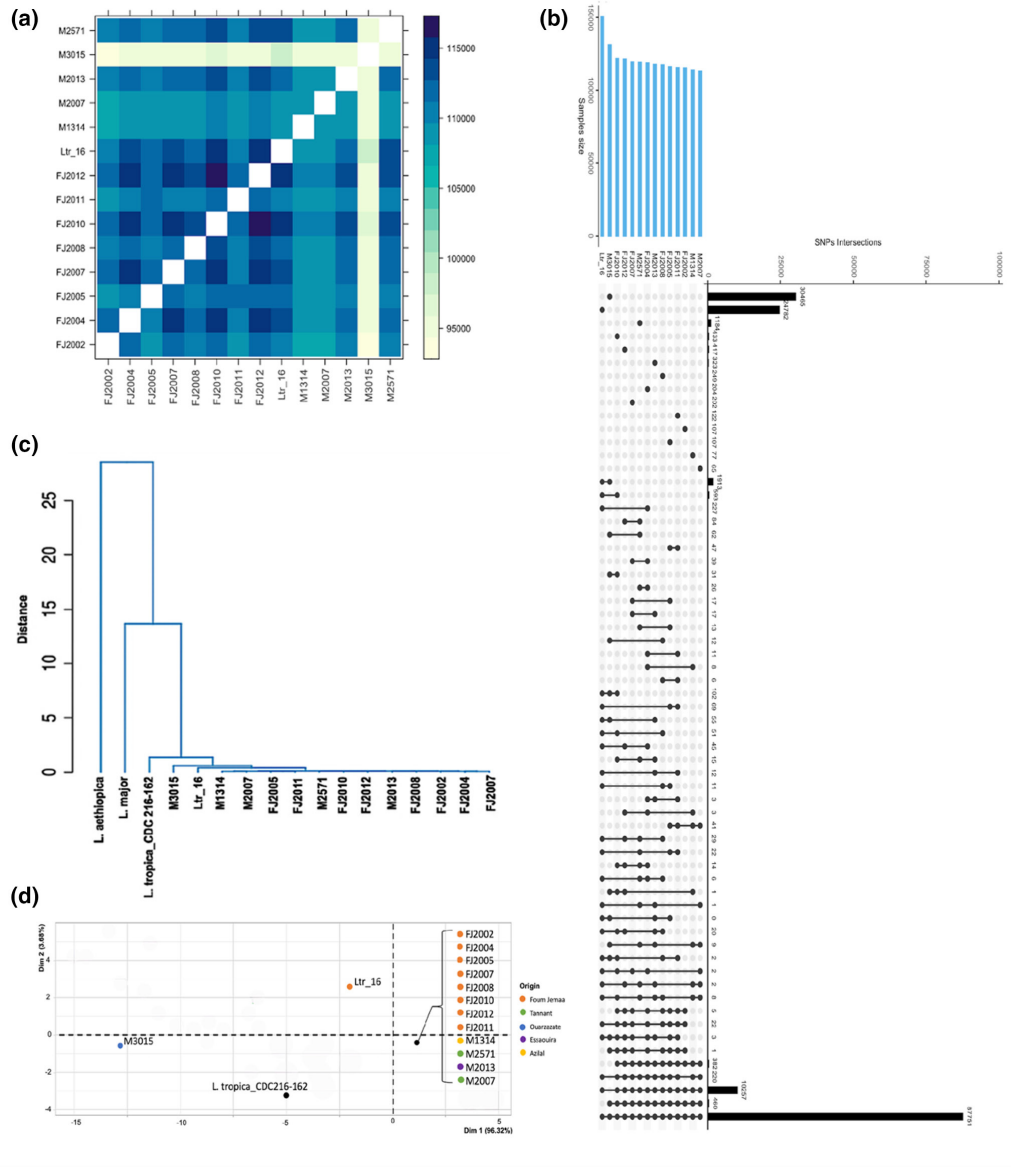
SNP analyses. (**a**) Heatmap visualizing the intersection of SNP numbers across all 14 samples. The diagonal cells (white) represent self-intersections. (**b**) UpSet plot illustrating shared and unique SNPs across all 14 isolates (selected comparisons are shown). (**c**) Dendrogram plot reconstruction with added reference genomes for *Leishmania major* and *Leishmania aethiopica* as outgroups. (**d**) PCA of the 14 isolates and the reference genome of *L. tropica*. The colour key identifies the geographical origin for each sample.

The heatmap in Fig. 4(a) represents the total number of common SNPs between pairs of isolates, providing further insight into their genetic relatedness. As judged by the colouring of the cells, M3015 is clearly the most diverse isolate as it shares the fewest SNPs with the other isolates, followed by M1314 and M2007. In contrast, isolates FJ2012 and FJ2010 shared the highest number of SNPs. The UpSet plot shown in Fig. 4(b) allows for a more fine-grained analysis of SNP distributions as it specifically indicates the number of SNPs that are shared exclusively between the indicated combinations of samples. From a total of 194 395 SNPs detected in the 14 strains, 87 751 SNPs (45.1 %) are shared among all samples, supporting their common evolutionary history. As expected, both diverse strains M3015 and Ltr_16 show the highest number of unique SNPs, i.e. 30 465 (15.7  %) and 24 782 (12.7  %), respectively, while only 1913 SNPs (1 %) are exclusively shared between these two strains. These substantial numbers of unique SNPs in both isolates underline their difference not only from each other, but also from the other 12 isolates.

Finally, we compared the genomic distance between the 14 *L*. *tropica* isolates using the corresponding giptools module that measures average nucleotide identity (ANI) scores between all sample pairs (see Table S6). This analysis included the *L. tropica* Leishmania_tropica_CDC216-162 reference genome, as well the genomes of *Leishmania aethiopica* (LaethiopicaL147) and *L. major* (LmajorFriedlin) as outgroups. Both the dendrogram (Fig. 4c) and PCA plot (Fig. 4d) confirm the genetic distance of the two diverse strains M3015 and Ltr_16 that cluster separately. These analyses further reveal a tight cluster of 12 isolates, which originate from all five sampled regions, suggesting the presence of a dominant *L. tropica* strain in Morocco with a signature SNP profile.

In conclusion, our data shed first light on the diversity of Moroccan *L. tropica* strains on the genomic level and identify a potential signature profile common to all samples, while at the same time revealing the coexistence of genetically diverse parasite populations. However, due to the limited sample size, these findings will need to be validated in future studies using larger sample sizes.

### Phylogenetic analysis of the Moroccan *L. tropica* isolates

**Fig. 5.**
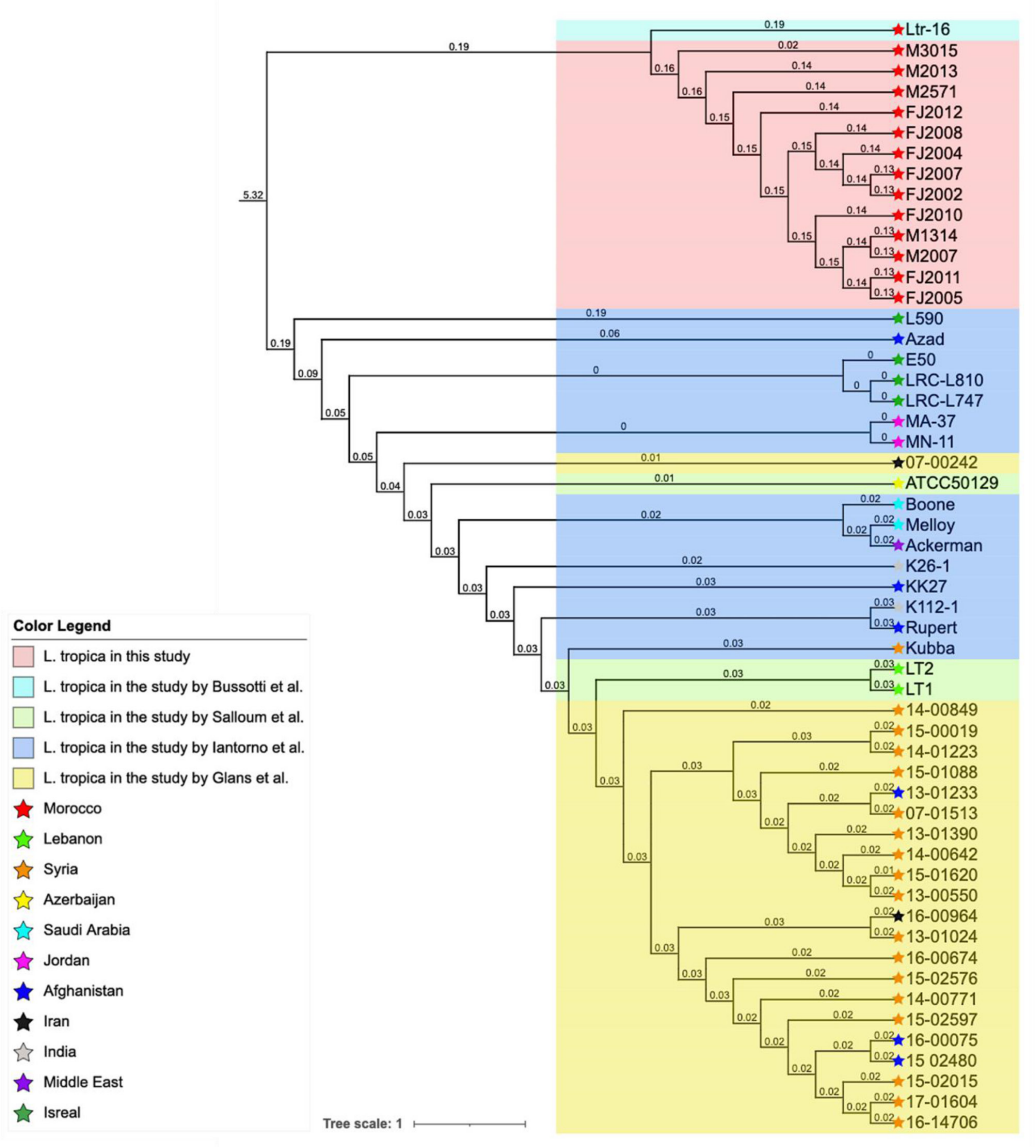
Phylogenomic tree analysis comprising 14 Moroccan *L. tropica* strains and 40 strains from a variety of geographical locations, including Lebanon, Syria, Azerbaijan, Afghanistan, Iran, Jordan, Saudi Arabia, India, the Middle East and Israel. The scale bar represents evolutionary distance measured in nucleotide substitutions per site.

We next performed phylogenomic analysis to substantiate the existence of a regional SNP profile among *L. tropica* isolates from Morocco and gain first insight into the possible origin of the divergent isolates Ltr_16 and M3015. This involved a comparative genomic investigation encompassing our cohort of 14 Moroccan isolates against a backdrop of 40 publicly available *L. tropica* genomes originating from diverse publications and an array of locations including Lebanon, Syria, Azerbaijan, Afghanistan, Iran, Jordan, Saudi Arabia, India, the broader Middle East and Israel [14,26, 33, 34] (see Fig. 5).

Our phylogenomic analysis reveals notable parallels to the results of previous phylogenetic studies. For example, our analysis reproduced the close genetic relationships between strains LT1 and LT2 shown by Salloum *et al*. [33], and between strains MN-11 and MA-37 shown by Iantorno *et al*. [26]. Likewise, in line with the observations made by Glans *et al*. [34], our analysis confirmed the close genetic relationship between isolates 15-02015, 16-14706 and 17-01604, as well as between isolates 16-00075 and 15-02480. Significantly, this comprehensive analysis revealed a phylogenetic cluster of the 12 Moroccan isolates that constituted the core group, thus corroborating our previous results obtained by PCA and dendrogram analyses (see Fig. 4). Surprisingly, the isolates Ltr_16 and M3015 were grouped in the same cluster, suggesting a possible shared ancestry between these divergent strains and the core group isolates, distinct from the 40 *L*. *tropica* strains of different geographical origin. This pronounced genetic departure among the Moroccan isolates from their international counterparts underscores a potential localized evolutionary trajectory, probably driven by unique ecological pressures that warrant further investigation.

## Discussion

Our study for the first time applied a comparative genomics approach on *L. tropica* isolates from Morocco to further assess the published hypothesis that this parasite species is genetically very heterogeneous in Morocco [6,7, 11]. SNP analysis of 14 *L*. *tropica* isolates revealed a genetically highly related core group of 12 isolates that showed notable divergence to the *L. tropica* reference genome generated from an Afghanistan isolate [17], and a series of publicly available *L. tropica* genomes from various countries across the Middle East. This suggests the presence of a predominant strain in Morocco that shows a region-specific SNP signature. We further identified two isolates (Ltr_16 and M3015) that diverged both from the reference genome as well as from the core group, revealing the existence of genetically divergent *L. tropica* strains in Morocco. Future sampling efforts and comparative genomic analyses will allow us to assess whether these two strains represent unique outliers or define coexisting sub-populations, as would be predicted by previous epidemiological studies that suggested a relatively high genetic diversity of *L. tropica* in Morocco [8,12].

*Leishmania* parasites have survived over many millions of years under selective pressures caused by natural ecological changes (storms, floods, hurricanes, volcanic eruptions, etc.), leading to frequent disruption of established host–vector relationships, which may explain today’s genotypic complexity of *Leishmania* vectors and reservoirs. Previous climatic changes, ice ages, the formation of arid regions, as well as landmass disruptions are probably responsible for the *Leishmania* genetic variability we observe today at the species level. This genetic variability has been shaped by genetic drift, natural selection and other evolutionary mechanisms, contributing to the divergence of organisms, populations and species. Little is known on how *Leishmania* responds to more local changes in ecology through short-term evolutionary processes – an open question of increasing importance given the accelerating ecological changes due to deforestation, global warming and armed conflicts [35]. Adaptation of *Leishmania* to these changes through its intrinsic genome instability may have unpredictable consequences on the clinical outcome and the epidemiology of the disease. The genomic divergence we have revealed in our study within the Moroccan samples as well as to strains from other middle eastern countries suggests that *L. tropica* could serve as an intriguing ecological model for future studies to assess the rapid adaptation processes occurring in *Leishmania* under field conditions.

In Morocco, the epidemiology of CL due to *L. tropica* is not yet fully understood. Although the epidemics caused by these parasites are less severe than those caused by *L. major*, *L. tropica* is perceived as a more important public health threat due to its wide geographical distribution [8,36, 37]. The genomic diversity we document in our study in isolates from Morocco and other endemic countries suggests a high level of *L. tropica* evolvability, which would reinforce the clinical threat posed by this *Leishmania* species. Our study resonates with previous investigations on the genetic diversity and heterogeneity of *L. tropica* isolates in Morocco. First, a study by Krayter *et al*. [38] used multilocus enzyme electrophoresis (to compare the microsatellite profiles of nine *L. tropica* strains isolated from human cases of CL in two different provinces of Morocco, which were further compared to 147 strains isolated from different geographical locations worldwide). The study found that *L. tropica* strains in each of the two Moroccan provinces separated into two phylogenetic clusters independent of their geographical origin, indicating a high degree of genetic heterogeneity, which was linked to the importation of pre-existing variants of *L. tropica* into Morocco rather than local, divergent evolution [38]. Second, El Hamouchi *et al*. [39] analysed genetic polymorphisms of *L. tropica* strains isolated from 125 CL patients, which showed a notable correlation between intraspecific *L. tropica* variants and geographical origins of the isolates, and revealed 13 distinct haplotypes, thus confirming the genetic heterogeneity of *L. tropica* in Morocco [39]. Finally, a study by EL Kacem *et al*. [12] applied MLST on 48 samples from CL patients from two different localities in Morocco, again indicating high genetic divergence between and among populations [12].

In conclusion, our study provides first genomic evidence for *L. tropica* genetic heterogeneity within and between endemic regions, which is probably the combined result of genetic drift and geographical adaptation, the latter likely to be driven by specific ecological factors related to transmission. Our results open important questions on the evolutionary forces and ecological constraints that drive region-specific adaptation of *Leishmania* in general and *L. tropica* in particular. Future studies applying our comparative genomics approach to a larger number of geographically distinct *L. tropica* isolates will need to integrate eco-epidemiological information on sand fly species and host reservoirs to identify gene–environment interactions that may influence the clinical outcome of the disease in a given region and the propensity of the parasite to develop drug-resistant phenotypes.

## supplementary material

10.1099/mgen.0.001230 Uncited Fig. S1.

10.1099/mgen.0.001230 Uncited Table S1.

## Abbreviations

ANI: average nucleotide identity
CL: cutaneous leishmaniasis
CNV: copy number variations
CNV: copy number variation
GIP: Genome Instability Pipeline
INDEL: insertion/deletion
ITS: intergenic spacer regions
L.: Leishmania
MLMT: multilocus microsatellite typing
MLST: multilocus sequence typing
NNN: Novy–MacNeal–Nicolle
PCA: principal components analyses
SNP: Single Nucleotide Polymorphism
VCF: variant call file
WGS: whole genome sequencing
