# Supplementary material for: A comparative genomics approach reveals a local genetic signature of Leishmania tropica in Morocco
Source: Microb Genom. 2024 Apr 5;10(4):001230. doi: 10.1099/mgen.0.001230 (PMC11092093; doi:10.1099/mgen.0.001230)
Supplement: Uncited Fig. S1. [file mgen-10-01230-s001.pdf]

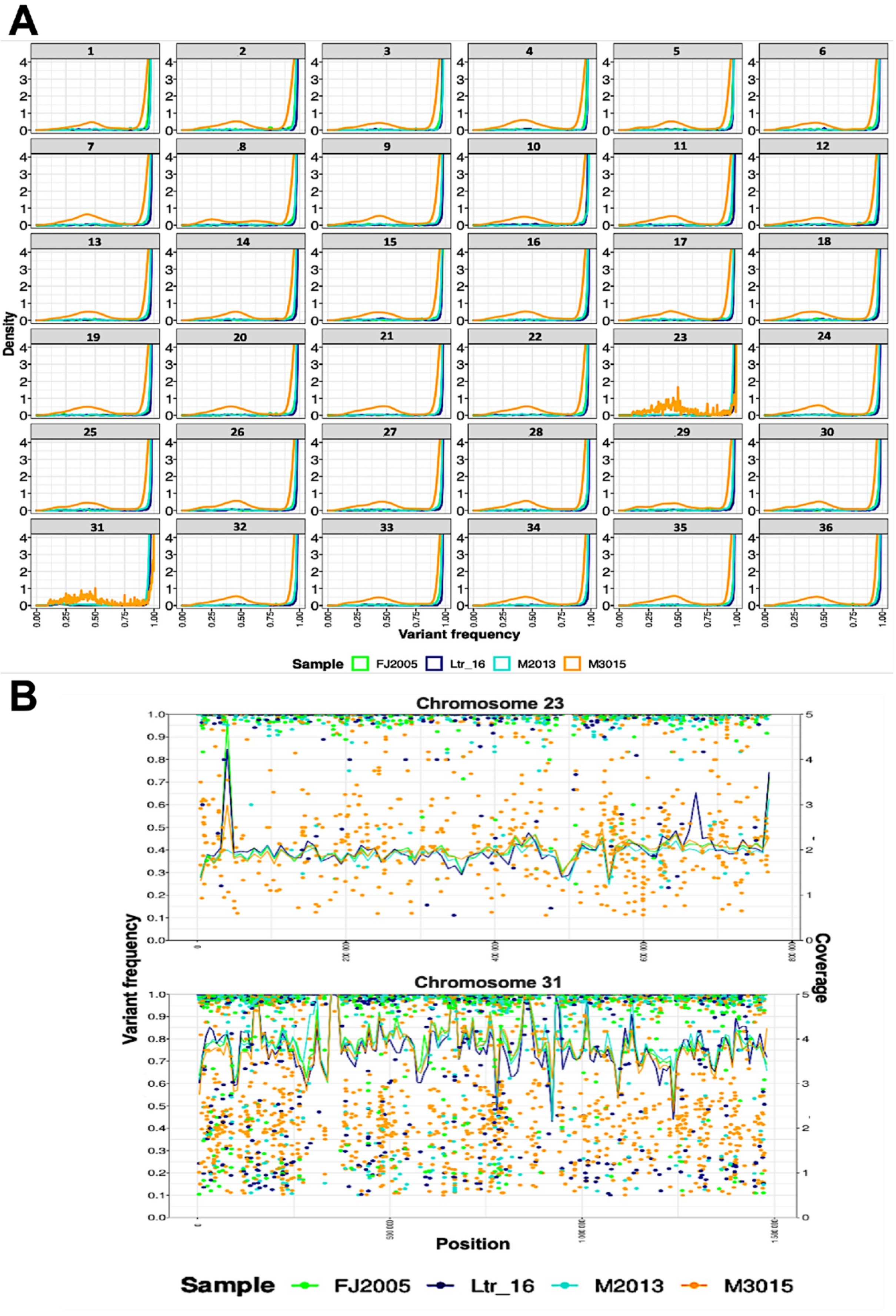

Figure S1: (A) SNP frequency density plots across the 36 chromosomes for M3015, Ltr_16, FJ2005, and M2013. The x-axis reports the variant allele frequency. The y-axis shows the estimated kernel density between 0 and 4. (B) SNP frequency density plots across the chromosome 23 and 31.
